# Development of a Plastic Embedding Method for Large-Volume and Fluorescent-Protein-Expressing Tissues

**DOI:** 10.1371/journal.pone.0060877

**Published:** 2013-04-05

**Authors:** Zhongqin Yang, Bihe Hu, Yuhui Zhang, Qingming Luo, Hui Gong

**Affiliations:** 1 Britton Chance Center for Biomedical Photonics, Huazhong University of Science and Technology-Wuhan National Laboratory for Optoelectronics, Wuhan, China; 2 MoE Key Laboratory for Biomedical Photonics, Department of Biomedical Engineering, Huazhong University of Science and Technology, Wuhan, China; University of Fukui, Faculty of Medical Sciences, Japan

## Abstract

Fluorescent proteins serve as important biomarkers for visualizing both subcellular organelles in living cells and structural and functional details in large-volume tissues or organs. However, current techniques for plastic embedding are limited in their ability to preserve fluorescence while remaining suitable for micro-optical sectioning tomography of large-volume samples. In this study, we quantitatively evaluated the fluorescence preservation and penetration time of several commonly used resins in a Thy1-eYFP-H transgenic whole mouse brain, including glycol methacrylate (GMA), LR White, hydroxypropyl methacrylate (HPMA) and Unicryl. We found that HMPA embedding doubled the eYFP fluorescence intensity but required long durations of incubation for whole brain penetration. GMA, Unicryl and LR White each penetrated the brain rapidly but also led to variable quenching of eYFP fluorescence. Among the fast-penetrating resins, GMA preserved fluorescence better than LR White and Unicryl. We found that we could optimize the GMA formulation by reducing the polymerization temperature, removing 4-methoxyphenol and adjusting the pH of the resin solution to be alkaline. By optimizing the GMA formulation, we increased percentage of eYFP fluorescence preservation in GMA-embedded brains nearly two-fold. These results suggest that modified GMA is suitable for embedding large-volume tissues such as whole mouse brain and provide a novel approach for visualizing brain-wide networks.

## Introduction

In recent years, plastic embedding has played an increasingly important role in three-dimensional, high-resolution imaging technologies [1]–[4]. To image large-volume samples with submicron or nanometer resolution, several well-established optical microscopy and electron microscopy imaging techniques have been combined with serial thin-sectioning to improve imaging depth or axial resolution [5]–[11]. In these studies, the biological samples are typically embedded in resin to meet the hardness required for thin sectioning.

Two alternatives currently exist for visually detecting a desired target in resin-embedded large-volume tissues: 1) staining of sections individually following resin embedding and serial thin-sectioning [7], [8] and 2) *en bloc* staining of tissue prior to resin embedding [5], [6]. The principal advantage of the section staining method is the wide availability of various histological staining techniques [12]. However, this method complicates the imaging process, particularly when a very large sample is imaged and thousands of consecutive sections require staining. In addition to its time-consuming and labor-intensive nature, staining also inevitably introduces slice deformation and subsequently leads to difficulties in three-dimensional registration and reconstruction of images. In contrast, *en bloc* staining circumvents the disadvantages of the sectioning staining technique. However, long staining incubations and limited staining methods for large-volume samples limit the utility of *en bloc* staining. A whole mouse brain, for example, requires staining with Golgi-Cox for 6 months [13]. Moreover, *en bloc* immunostaining of large-volume samples is not available due to the limited penetration depth of antibodies [14].

Genetically encoded fluorescent proteins are powerful markers for labeling specific proteins or cell types independent of tissue volume, and a great number of transgenic mouse lines have already been generated [15]. However, direct imaging of resin-embedded tissue expressing fluorescent proteins is difficult because the chemical reagents and high temperature conditions used during the process of plastic embedding may reduce fluorescence intensity [16]–[18]. Thus, when utilizing fluorescent protein-expressing samples for resin embedding-based, three-dimensional, high-resolution imaging techniques, it is of particular importance to preserve the fluorescence intensity of fluorescent proteins during plastic embedding.

To date, several researchers have attempted to detect fluorescent proteins in sections embedded with glycol methacrylate (GMA) [19], LR White [20]–[22], LR Gold [23], Lowicryl [24], [25] and JB-4 [26] resins (see review in Table 1). Despite published techniques and use by several laboratories, these methods have important limitations that must be considered. First, the commonly observed loss of fluorescence intensity during resin embedding is rarely quantified. Second, the samples used in these methods are generally very small, usually at the micrometer or millimeter scale. Whether these resins may also be utilized for embedding large-volume tissues such as whole mouse brain at the centimeter scale is unclear.

**Table 1:** Review on resins used for embedding samples expressing fluorescent protein.

Resins	Samples	Fluorescence labeling	Fluorescence preservation	References
GMA	C.elegans	Citrine & tdEos	70%	[19]
LR White	mouse brain section	YFP	—	[20]
	culture cells	GFP & YFP	—	[22]
	Zebrafish head	GFP	—	[21]
LR Gold	C.elegans	GFP & mRFP	—	[23]
Lowicryl	culture cells & yeast	GFP & mCherry	—	[24]
	Zebrafish embryos	GFP	—	[25]
JB-4	Zebrafish embryos	GFP	—	[26]

We set out to establish a new plastic embedding method that was simultaneously suitable for fluorescence preservation and the processing of large-volume tissues. In the current study, we quantitatively analyzed the fluorescence preservation capacity and penetration ability, which are essential for large-volume tissue preparation, of four commonly used hydrophilic resins including GMA, LR White, hydroxypropyl methacrylate (HPMA) [27] and Unicryl [28]. We utilized Thy1-eYFP-H transgenic mouse brains as the large-volume tissue sample [29]. To the best of our knowledge, this is the first report to systematically evaluate the fluorescence preservation and penetration abilities of different resins. Furthermore, we demonstrate that optimizing the formulation of GMA resin—by reducing the polymerization temperature, removing the inhibitor 4-methoxyphenol (MEHQ) and adjusting the pH to be alkaline—further improves its fluorescence preservation capacity.

## Results

### Fluorescence preservation of different resins

Currently, there are multiple types of commercially available resin embedding media. These agents can be roughly divided into two groups: hydrophobic resins (e.g., Spurr [30], Araldite [31] ) and hydrophilic resins (e.g., the resins in Table 1). Because fluorescent proteins maintain their conformation in hydrated states, hydrophilic resins are more likely to preserve fluorescence. In this study, we quantitatively examined the fluorescence preservation of four hydrophilic resins: GMA, LR White, HPMA and Unicryl.

The procedure for quantitative analysis of fluorescence preservation capacity of different resins was as follows. First, a Thy1-eYFP-H transgenic mouse brain was fixed with 4% paraformaldehyde. Second, 100-µm brain sections were prepared from the fixed brain. Third, two-photon microscopy was used to image neurons expressing eYFP, and the locations of the imaged neurons were recorded. Fourth, sections were processed with four resins. Fifth, sections were embedded between two coverslips. Finally, neurons expressing eYFP were reimaged by two-photon microscopy using the same parameters designated in step three. ImageJ software was used to analyze changes in the average fluorescence intensity of neurons between step three and the final step.

The results are shown in Table 2. Following embedding, incubation with HPMA resin doubled the fluorescence intensity of neurons expressing eYFP compared with pre-embedding, while GMA, Unicryl and LR White quenched fluorescence intensity to different extents. Most significantly, LR White quenched over 70% of eYFP fluorescence intensity. As reported previously, the fluorescence intensity of fluorescent proteins is related to the pH of the buffer solution, with a pH>7 being favorable for fluorescence preservation [18]. Our experiments revealed that the pH values of the four resins were HPMA>GMA>Unicryl>LR White, and that the pH value was positively correlated with the fluorescence preservation ability of each resin. This result may suggest that resin pH plays an important role in preserving fluorescence during plastic embedding.

**Table 2:** Comparison of fluorescence preservation and penetration ability of four resins.

Resins	pH^a^	Fluorescence preservation of neurons^b^	Time for penetration of a whole mouse brain
HPMA	7.12	210%	>2 weeks
GMA	6.0	69.98%	3 days
Unicryl	5.12	51.54%	2 days
LR White	4.8	27.59%	2 days

a the pH of four resins is tested with pH meter.

b Fluorescence preservation of a neuron = fluorescence intensity of neuron after embedding/fluorescence intensity of neuron before embedding * 100%. For each resin, at least fluorescence preservation of 6 neurons are analyzed and then averaged.

### Penetration ability of different resins

We tested the penetration ability of the four resins using whole mouse brain. The procedure was as follows. First, whole mouse brains fixed with 4% paraformaldehyde were prepared. Second, whole brains were dehydrated in a graded series of ethanol solutions and subsequently infiltrated in a graded series of resin solutions (up to a concentration of 100% resin). Third, the whole brains were embedded and polymerized after being immersed in the final 100% resin solution for 1, 2, 3, 5, 7, or 14 days. Finally, embedded whole brains were serial sectioned at 1-µm thickness and imaged using the Micro-Optical Sectioning Tomography (MOST) system [5] developed by our laboratory. Sections without folds or hollows were considered to have complete resin penetration.

The results in Table 2 demonstrate that the ability of HPMA to penetrate whole mouse brain was inferior to other resins, and a two-week incubation in HPMA solution was insufficient to penetrate the entire brain. The penetration speeds of GMA, Unicryl and LR White were much faster than that of HPMA, and complete penetration of whole mouse brain using these alternate resins was achieved within 2–3 days.

Because the penetration ability of GMA was superior to that of HPMA and the fluorescence preservation capacity of GMA was enhanced compared with Unicryl and LR White, we attempted to optimize the formulation of GMA to further improve its fluorescence preservation.

### Optimization of GMA resin

As reported previously, fluorescent proteins are temperature sensitive [32], and lower temperatures are favorable for reducing fluorescence loss during resin polymerization. The manual provided in the GMA water-soluble embedding kit recommended polymerizing the resin at 60°C. However, our experiments indicated that GMA was fully polymerized at 55°C if the pre-polymerization of GMA and sealing of the embedding mold were optimized. Thus, we set the sample polymerization temperature to 55°C (Group 2 in Table 3) and found that the fluorescence preservation was improved 11.78% relative to the original formulation (Group 1 in Table 3).

**Table 3:** Different formulation of GMA and its fluorescence preservation.

Test factors	Group 1	Group 2	Group 3	Group 4	Group 5	Group 6
Polymerization temperature	60°C	55°C	55°C	55°C	55°C	55°C
Remove inhibitor MEHQ	NO	NO	YES	YES	YES	YES
Volume of Ethanolamine	0	0	0	0.1‰	0.5‰	1‰
Fluorescence preservation^a^	69.98%	81.76%	112.02%	123.2%	120.91%	—

a For each group, mean value of fluorescence preservation of 6 neurons is calculated.

To prevent resin polymerization during long-distance transport, MEHQ is included in the GMA embedding kit along with the GMA monomer and n-butyl methacrylate. During the embedding process, however, MEHQ may change the conformation of fluorophores and lead to fluorescence quenching. Therefore, we removed MEHQ from the GMA kit using an A1_2_O_3_ column prior to incubating brain samples in the resin (Group 3 in Table 3). With this modification, the fluorescence preservation capacity of GMA was improved by 30.26% compared with the formulation indicated in Group 2.

Previous studies have shown that the fluorescence intensity of fluorescent proteins decreases as the acidity of the environment increases [18]. In our hands, the typical pH value of GMA was approximately 6.0, although the precise pH value varied depending on the brand (Ted Pella, SPI, Electron Microscopy Sciences, et al.) and batch of the GMA embedding kit. Therefore, we adjusted the pH of GMA to be alkaline using various volumes of 25% ethanolamine solution (Group 4–6 in Table 3). The results demonstrated that fluorescence preservation using the pH-adjusted GMA in Group 4 and Group 5 was improved by 11.18% and 8.89%, respectively, compared with the formulation indicated in Group 3. The fluorescence preservation of Group 6 GMA was not quantified due to the poor polymerization quality of the resin, which may suggest that an excessively alkaline pH disrupts the polymerization of GMA resin.

**Figure 1:**
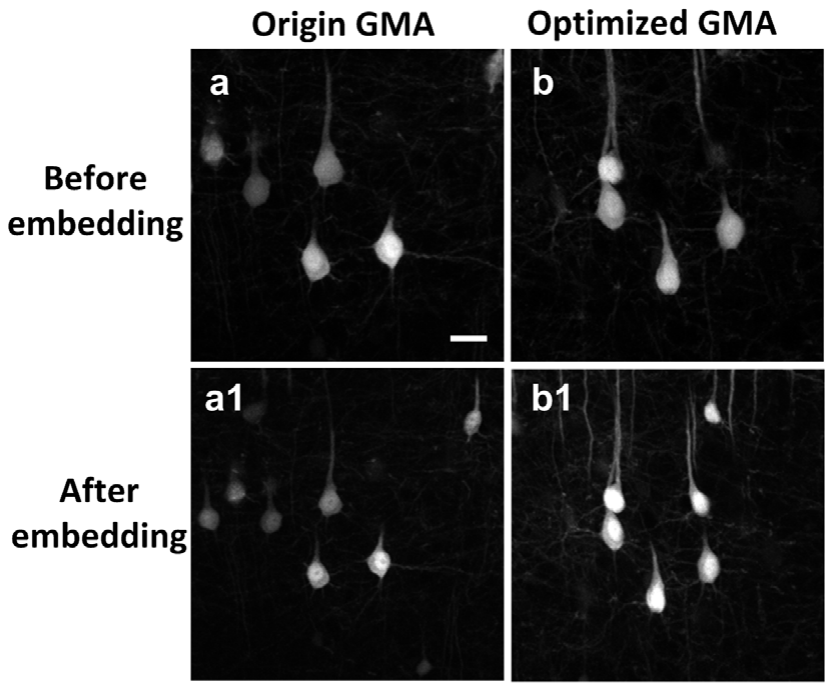
Fluorescence intensity of neurons is improved with optimized GMA resin. Brain sections imaged with two-photon microscopy using the same parameter settings before (a, b) and after embedding (a 1, b 1). Scale bar, 20 µm. Each image is a max-projection of the image stacks of the brain slice (thickness: 20 µm).

**Figure 2:**
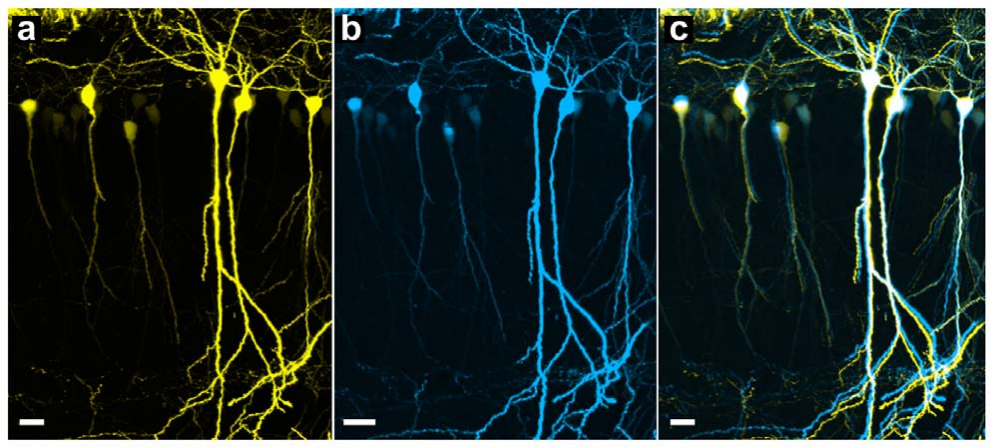
Morphology of pyramidal neurons (hippocampus) is well preserved after being embedded in optimized GMA resin. A brain section from a Thy1-eYFP-H mouse imaged with two-photon microscopy before (a) and after embedding (b). (c) Merged images from (a) and (b). Scale bar, 20 µm. Each image is a max-projection of the image stacks of the brain slice (thickness: 60 µm).

Following the modification of the GMA resin as described above, the fluorescence preservation of eYFP-labeled neurons using the optimized GMA solution improved nearly two-fold (Table 3 and Figure 1). To evaluate preservation of structural morphology in brains incubated with the new formulation of GMA, we imaged 100-µm brain sections before and after embedding. The results presented in Figure 2 demonstrate that neuronal morphology also remained intact using the optimized GMA formulation. After embedding, the shrinkage of 100-µm brain sections in X- and Y- direction was 15.4% and 15.8%, respectively.

## Discussion

Embedding samples in resin followed by continuous thin-sectioning and imaging is an effective way to obtain three-dimensional, high-resolution data from large-volume tissues. However, available applications of this approach for large-volume tissues expressing fluorescent proteins are currently limited. To establish a plastic embedding method that is suitable for embedding large-volume samples expressing fluorescent protein, we quantitatively studied the fluorescence preservation and penetration abilities of four resins using Thy1-eYFP-H transgenic mouse brain.

To the best of our knowledge, there has been no previous report regarding the detection of fluorescent protein in samples embedded in HPMA or Unicryl resin. Our findings demonstrated that HPMA resin significantly improves the fluorescence intensity of eYFP-labeled neurons but has poor penetration into whole brain. Unicryl resin rapidly penetrates whole mouse brain but results in a 50% loss of eYFP fluorescence intensity. LR White resin has been applied to a variety of tissue samples expressing fluorescence protein [20]–[22], although the effect of LR White on fluorescence quenching was not previously quantified. In our study, LR White poorly preserved fluorescence, reducing eYFP fluorescence intensity by over 70%. Thus, detecting fluorescence signals in LR White-embedded sections necessitates samples that exhibit high expression of fluorescent proteins and the use of intense excitation light sources. GMA has also been reported as a suitable resin for embedding roundworms expressing fluorescent protein [19], with GMA preserving 70% of fluorescence intensity in samples following embedding. Our results using GMA embedded mouse brain were consistent with the previous study, as we observed 69.98% eYFP fluorescence preservation.

Because GMA was suitable for embedding large-volume samples, we further optimized the formulation of GMA resin to improve its fluorescence preservation capacity. Fluorescence intensity of many fluorescent proteins is temperature - and acid - sensitive and may be decreased by the inhibitor MEHQ contained in the GMA embedding kit. Accordingly, we optimized the GMA embedding solution by reducing the polymerization temperature, removing MEHQ and adjusting the solution pH to be alkaline. These modifications of the GMA formulation improved fluorescence preservation of eYFP-labeled neurons nearly two-fold while also maintaining neuronal morphology.

In summary, we have developed a GMA embedding method that allows for embedding of large-volume samples with significant enhancement of fluorescence intensity. With the latest developments in three-dimensional fluorescence imaging technology based on continuous thin-sectioning, the development of high-resolution databases of large-volume samples is promising. In future studies, we hope to combine a microwave processing method to improve the penetration ability of HPMA resin, as the fluorescence preservation capacity of HPMA is ideal for fluorescence imaging studies.

## Methods

### Preparation of brain sections

Eight-week-old Thy1-eYFP-H transgenic mice (Jackson Laboratory, USA) were used in this study. Mice were anesthetized with a 1% solution of sodium pentobarbital and intracardially perfused with 0.01 M phosphate buffered saline (PBS, Sigma) followed by 4% paraformaldehyde (Sigma-Aldrich) in 0.01 M PBS. The entire brain was removed and post-fixed in 4% paraformaldehyde at 4°C for 24 h. After fixation, the mouse brain was rinsed overnight at 4°C in a 0.01 M PBS solution, and 100-µm brain sections were prepared with a vibratome (Leica VT 1000s). Animal care and use was done in accordance with the guidelines of the Administration Committee of Affairs Concerning Experimental Animals in Hubei Province of China. The protocol was approved by the Committee on the Ethics of Animal Experiments of the Huazhong University of Science and Technology (Permit Number: 00027340). All surgery was performed under sodium pentobarbital anesthesia, and all efforts were made to minimize suffering.

### Resin processing of brain sections

Before resin infiltration, the 100-µm brain sections were subsequently dehydrated in 50%, 70%, and 95% ethanol solutions for 5 min each. After dehydration, sections were subsequently infiltrated with 70%, 85% and two rinses of 100% resin solution for 5 min each, followed by 100% resin solution for 2 h. All dehydration and infiltration steps were carried out at 4°C. The 70% and 85% resin solutions were prepared with 95% ethanol solution.

### Embedding and polymerization of the brain sections

First, a section was mounted on a 24×50 mm^2^ microscopy coverslip, and a small amount of resin was dropped onto the section. Next, the section was covered with another coverslip, while ensuring no air bubbles were present between the two coverslips. Finally, the embedded section was placed into an oven and polymerized for 6 h.

### Sectioning and imaging

A two-photon fluorescence microscopy (FV-300, Olympus, Japan; Chameleon Ultra II, Coherent; 40× water-immersion objective, NA 0.8) was used to image the 100-µm brain sections, by which serial optic slices were obtained and then projected images were make. For resin-embedded whole mouse brain, a customized microtome named Micro-Optical Sectioning Tomography (MOST) system [5] was used to make 1-µm sections and image the sections at the same time.

### Fluorescence quantification

Fluorescence intensity of neurons in images was analyzed using ImageJ software. First, using the rectangular-selection tool, a rectangle area was selected in the soma of a neuron. Next, using the histogram tool, the mean fluorescence intensity of the rectangle area was measured, which was served as the fluorescence intensity of the neuron.

Suppose the fluorescence intensity of a neuron before embedding is “A”, after resin embedding, its fluorescence intensity was changed to “B”. We calculated “fluorescence preservation of a neuron during embedding = 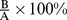
”. For each experiment group, mean value of fluorescence preservation of 6 neurons was calculated.

### Components of the four resins

The components of the HPMA (SPI) solution were 100 g HPMA and 0.1 g benzoyl peroxide. The components of the GMA (Ted Pella) solution were 67 g GMA (contains few MEHQ), 3 g distilled water, 30 g n-butyl methacrylate (contains few MEHQ) and 0.6 g benzoyl peroxide. The components of LR White (Medium Grade, Ted Pella) were 500 ml LR White and 9.9 g benzoyl peroxide. Unicryl (SPI) is provided as a single solution and can be used directly.
